# Segmental Absence of Intestinal Musculature in a Child with Type IV Ehlers–Danlos Syndrome

**DOI:** 10.3390/children8080680

**Published:** 2021-08-05

**Authors:** Nicole Zeky, Celia Short, Brent Keith, Randall D. Craver, Jessica A. Zagory

**Affiliations:** 1 Department of Pediatrics, Division of Gastroenterology, Louisiana State University Health Sciences Center, New Orleans, LA 70112, USA; nzeky@lsuhsc.edu (N.Z.); bkeith@lsuhsc.edu (B.K.); 2 Department of Surgery, Louisiana State University Health Sciences Center, New Orleans, LA 70112, USA; cshor4@lsuhsc.edu; 3 Department of Pathology, Louisiana State University Health Sciences Center, New Orleans, LA 70112, USA; randall.craver@lcmchealth.org; 4 Department of Surgery, Division of Pediatric Surgery, Children’s Hospital New Orleans, 200 Henry Clay Avenue, New Orleans, LA 70118, USA

**Keywords:** Ehlers–Danlos, vascular Ehlers–Danlos, intestinal perforation, segmental absence of intestinal musculature

## Abstract

Patients with vascular Ehlers–Danlos syndrome (vEDS) have a defect in the formation of type III collagen. This defect puts patients at risk of vascular rupture, uterine rupture, and bowel perforations. The segmental absence of intestinal musculature is a rare histopathologic finding, wherein there is a lack of a muscularis propria layer in the intestinal wall. Although typically documented in the literature in neonates or adults, it can be seen in children of other ages. This is a case report of a patient who exhibits both rare entities, which has not been described in the literature to date.

## 1. Introduction

The segmental absence of the intestinal musculature (SAIM) is a rare clinicopathological entity characterized by the partial or complete absence of the muscularis propria and is associated with intestinal obstruction and perforation [1,2,3]. SAIM was first described in 1967 by Emanuel et al. as a cause of neonatal intestinal obstruction [4]. Patients with a segmental absence of intestinal musculature present with abdominal distension, diminished bowel sounds, and eventual bowel obstruction or perforation [1,3,5]. This results in a lack of peristalsis and dilation of the proximal segment of the bowel [5]. To date, fewer than 50 cases of SAIM have been reported in the English literature. While the majority of cases are reported in the neonatal age group, reports of adolescent and adult SAIM have also been described [1,6,7,8].

Although the true pathophysiology of SAIM is unknown, theoretical classifications of the disease include primary idiopathic and secondary acquired [1,2,3]. Primary idiopathic is hypothesized to be secondary to abnormal embryogenesis with incomplete or discontinuous myogenesis [9] or from developmental diverticula in the embryonic small bowel, causing gaps in the muscularis propria [10]. In these cases, there appears to be no inciting events and the histology demonstrates only a loss of the muscularis propria layer without pathologic findings in the remaining layers, such as fibrotic or inflammatory infiltrates [2]. In contrast, secondary acquired SAIM is proposed to result from an ischemic event either in utero or postnatally, in which both the mucosa and muscularis propria are injured. With the relatively high regenerative capacity of the mucosa, the mucosa is repaired, leaving the exclusive absence of the muscular layer [2,3,11]. There are no surrogate biomarkers, to date, for patients suspected of SAIM, as this is made on biopsy alone.

Herein, we present a case of perforated SAIM in a patient with newly diagnosed vascular Ehlers–Danlos syndrome (vEDS). Although vEDS is a relatively rare condition, spontaneous intestinal perforation is a well-documented complication of the disease [9]. With our current findings, secondary SAIM may be an unrecognized entity in vEDS patients presenting with spontaneous intestinal perforation.

## 2. Case Reports

A previously healthy 16-year-old female presented with left lower quadrant abdominal pain that had acutely intensified reported episodes of abdominal pain over the last two years prior to presentation. Her stools alternated between constipation and diarrhea, and she was previously diagnosed with irritable bowel syndrome. Her pain on presentation was severe and was associated with non-bloody, non-bilious emesis.

On admission, she was afebrile but diaphoretic and tachycardic with a heart rate of 150, which improved with fluid resuscitation. Her abdomen was soft but tender throughout, with voluntary guarding on palpation on the left and hypoactive bowel sounds. A rectal exam demonstrated a good rectal tone and no stool in the rectal vault.

**Figure 1:**
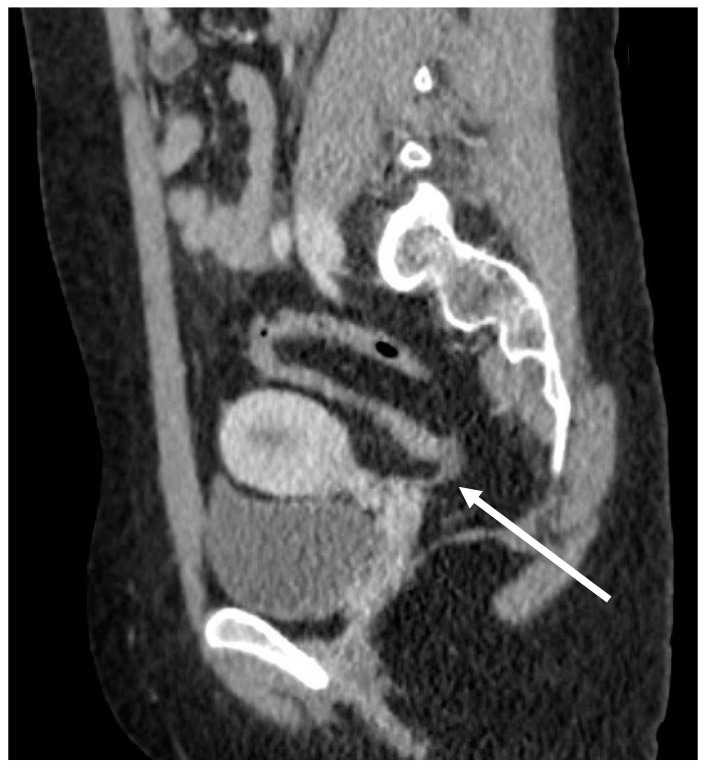
Sagittal view of an abdominal CT scan from admission showing sigmoid colon narrowing (arrow).

Initial laboratory investigation included urinalysis, complete blood count, and basic metabolic panel and were unremarkable. An abdominal CT was notable for a stool burden through the distal left colon and to the mid sigmoid with collapsed distal sigmoid and rectum, for which Hirschsprung’s disease was suggested as a differential diagnosis (Figure 1). There was also fat stranding around the sigmoid, which added inflammatory bowel disease to the differential. She was started on intravenous hydration and broad-spectrum antibiotics for a possible infectious process.

On hospital day 2, she developed a fever and right shoulder pain, prompting an abdominal X-ray that showed pneumoperitoneum. She was taken to the operating room for an exploratory laparotomy. She was found to have a large perforation in the sigmoid within a necrotic segment and fecal peritonitis. Friable mesentery and surrounding tissue was also noted. Her sigmoid colon was resected to grossly healthy tissue and a colostomy was created.

The patient recovered from the operation after a short stay in the intensive care unit. On further review of her history, it was found that she often experiences joint dislocation in the hips, although her siblings do not. There was also previously unknown family history of elastic skin or joint dislocations in the maternal grandmother.

**Figure 2:**
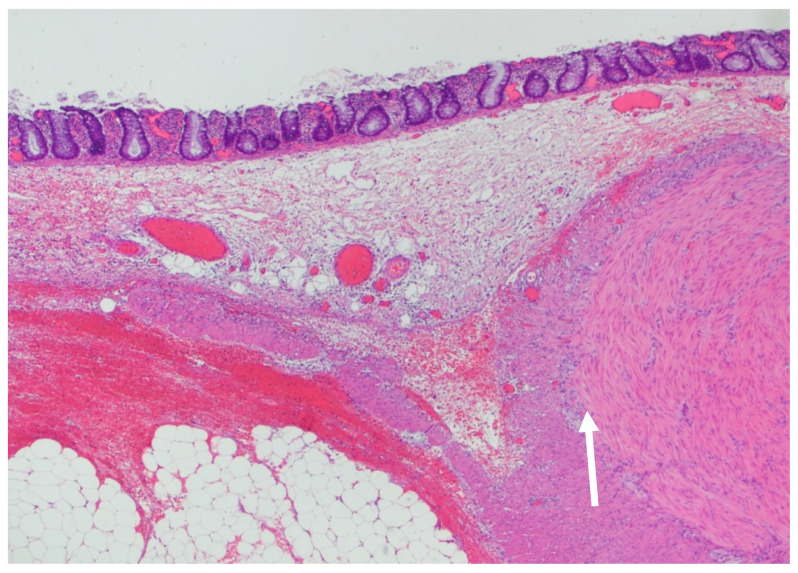
Section of the colon that demonstrates the inner muscular layer ending abruptly (arrow). The outer muscular layer is split, the inner portion wrapping around the blunt end of the inner muscular layer. Myenteric (Auerbach’s) plexus is visible between this portion of the outer muscular layer and the abruptly terminating inner muscular layer. The outer portion of the outer muscular layer continues, becoming attenuated and finally ending. The muscularis mucosa, immediately below the lamina propria containing normal colonic glands, is present throughout (Hematoxylin and Eosin, 20×).

**Figure 3:**
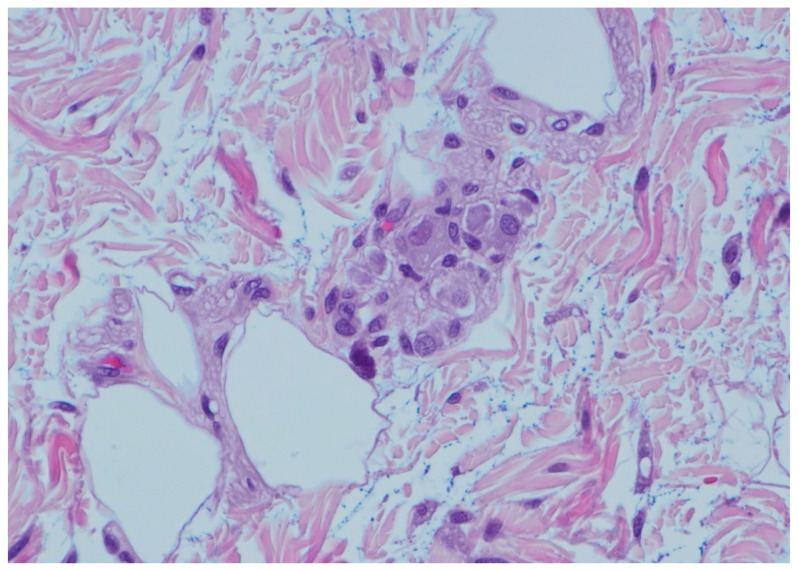
Giant submucosal ganglion with at least 8 ganglion cells. Twenty percent of the submucosal ganglion had at least 8 ganglion cells. The ganglia were often located in the middle of the submucosa, between the Meissner and Henle plexi (Hematoxylin and Eosin, 400×).

On histopathology, there was a loss of the muscularis propria consistent with SAIM, with preservation of the muscularis mucosa, which was more prominent near areas of the perforation (Figure 2). There was noted to be a good transition from normal colonic musculature to areas absent of an inner muscular layer. Areas of the tissue showed full-thickness necrosis and mucosal exudate, with other dusky areas noted of marked vascular congestion. Ganglion cells were found in both the submucosal plexus and in the muscularis propria, which is an abnormal variant since these are often found in between these two layers (Figure 3).

Due to intraoperative findings of the friable mesentery and fragile colonic tissue, there was concern about a connective tissue disorder. Our patient demonstrated characteristic facies of EDS, with translucent skin, thin vermilion of the lips, and a narrow nose. There was no documented family history of any connective tissue disorders. Genetic testing resulted with a heterozygous mutation of COL3A1 with a pathogenic variant of the c.1511 defect consistent with vEDS. Cardiology was consulted and carried out an echo, showing mild aortic root dilation.

## 3. Discussion

EDS is a connective tissue disorder characterized by hypermobile joints, easy bruising, and fragile skin, but can also include changes in stature, clubfoot, small chin, mitral valve prolapse, and spontaneous pneumothorax [12,13,14]. It is an overarching disease with several subtypes, all of which have defective collagen formation [12]. Gastrointestinal issues are common in patients with all subtypes of EDS, including irritable bowel syndrome, dysphagia, gastroesophageal reflux disease, constipation, hiatal hernias, and diverticulosis [12].

One subtype of EDS, classified as the vascular type, is due to a defect in type III collagen [13,14]. It is the rarest of the different types and occurs in approximately one in 100,000 live births. Diagnosis is usually made after a major complication, including a bowel perforation or blood vessel perforation [13,14,15]. The pattern of inheritance is autosomal dominant and consists of a genetic defect in the COL3A1 gene (2q32.2), which encodes for the pro-alpha1 chains of type III procollagen [13,14,15]. Type III collagen is an important component of the mechanical strength of hollow structures, including the bowel [15].

A bowel perforation is a known complication in patients with vEDS, but usually occurs later in life with an average age of 21 years [13,14]. Colonic perforation, more specifically the sigmoid colon, is the most common place for spontaneous rupture of the gastrointestinal tract [13,14,15,16,17]. A review of 220 patients with confirmed vEDS showed that 40% had a bowel perforation, most of which consisted of perforation in the sigmoid colon [14]. One case report of a six-year-old boy indicated that he had two bowel perforations—one initially in the colon with a colostomy placed and then another perforation proximal to the colostomy site [13]. Despite being at an increased risk, one study found that approximately 50% of patients did not experience a second bowel perforation after their first [15]. Post-operatively, patients with vEDS are at higher risk for complications secondary to easy bleeding and poor wound healing [18,19].

Mortality for vEDS patients falls under their risk for visceral or vascular perforation, including aortic rupture, uterine rupture, and bowel perforation [19]. Approximately 25% of patients will have some form of complication like this by the time they are 25 years old [19]. One systemic review of colonic complications in vEDS included 48 patients who underwent 102 operations [19]. Most were emergent colonic perforations (98%) and half of those patients had repeat bowel perforations on average just 12 months after their first surgery [19].

There are no clear guidelines on the management of these patients or on preventing their risk of recurrent bowel perforation [13]. Constipation is something that patients with EDS suffer with already, and a good bowel regimen might help lower the risk of bowel perforation [13]. Long-term risks include arterial tear, major blood vessel perforation, and uterine rupture during childbirth [14].

Patients with SAIM present with abdominal distension, diminished bowel sounds, and eventual bowel obstruction or perforation [1,3]. It results in a lack of peristalsis and dilation of the proximal segment of the bowel [1,3]. The underlying etiology is unclear; however, cases reported in the literature seem to show several cases occurring in premature infants [1,3]. Davis et al. published a retrospective review of surgical pathology from 2003 to 2010 with noted SAIM [3]. Data were pulled from both the mother’s medical history, as well as the pediatric patient [3]. Five pediatric patients were identified, all of whom were born premature between 25 and 32 weeks of gestation, and four of them had a patent ductus arteriosus (PDA), with two receiving indomethacin [3]. There is some thought that because SAIM is associated with premature infants with PDAs, the underlying etiology on SAIM is tied to ischemia [3]. This report hypothesizes that ischemia can occur but is transmural, and the mucosa regenerates more quickly after adequate blood flow is restored [3]. The muscular layer lags in repair, leading to an absence found in the histopathology [3]. It is possible that the vascular and collagen abnormalities in vEDS may lead to a similar finding.

Cases of SAIM are more commonly documented in the literature in the neonate and adult population; however, there have been reports of it in older children and adolescents, such as our patient here. One case report documented a four-year-old who initially presented with imaging concerning inflammatory bowel disease and small bowel wall thickening leading to concerns of stenosis [1]. Pathology and endoscopy showed normal mucosa [1]. Ultimately, the patient was referred for surgery, and a prominently dilated small bowel proximal to the ileocecal valve was reported upon laparoscopy [1]. The resected segment was noted to have incomplete muscular arrangement and replacement of the muscular layer with fat, consistent with SAIM [1]. Another case report documented a 10-year-old male who presented with an acute abdomen and peritonitis [7]. He underwent a right hemicolectomy and partial resection of the ileum, with the pathology showing a lack of the muscularis propria layer [7].

Although it is well documented that patients with vEDS are at risk of intestinal perforation, the link between vEDS and SAIM is not, to the best of our knowledge, explicitly documented in the literature thus far. Type III collagen is an important structural protein for the integrity of blood vessels, the uterus, and the bowel wall [18]. It also plays a role in cell adhesion and cell migration [18]. A lack of a musculature layer leads to poor peristalsis and increased luminal stress on the intestinal wall [1]. Our patient’s additional lack of properly formed collagen in the bowel wall put them at greater risk of perforation. An interference of cellular migration might be the link between these two rare conditions in our patient.

## 4. Conclusions

vEDS patients are at high risk of vascular, uterine, and intestinal perforations. Due to the type III collagen defects of this condition, it is possible that there is a link between this rare disease and another rare entity, SAIM, due to defects in cell adhesion and migration.

## Data Availability

No data.
